# Psychometric evidence for assessing early childhood development in Brazil: the Child Development Diagnostic Form in the Criança Feliz Program

**DOI:** 10.1136/bmjpo-2025-004362

**Published:** 2026-08-04

**Authors:** Ellayne Souza Cerqueira, Michael E Reichenheim, Ana Angélica Rodrigues Alves, Ricardo Rodrigues Dutra, Claudia Leite Moraes, Priscila Ribas de Farias Costa, Rita de Cássia Ribeiro-Silva, Mauricio L Barreto

**Affiliations:** 1 Post-Graduation Program in Food, Nutrition and Health, School of Nutrition, Federal University of Bahia, Salvador, Brazil; 2 Center for Data and Knowledge Integration for Health (CIDACS), Gonçalo Moniz Institute (IGM), Oswaldo Cruz Foundation (Fiocruz), Salvador, Bahia, Brazil; 3 Department of Epidemiology, Institute of Social Medicine, Rio de Janeiro State University, Rio de Janeiro, RJ, Brazil; 4 United Nations Development Programme, Brasília, Distrito Federal, Brazil; 5 Ministry of Development and Social Assistance, Family and Fight against Hunger, Brasília, Distrito Federal, Brazil

**Keywords:** Epidemiology, Child Health, Children, Developing Countries, Low and Middle Income Countries

## Abstract

**Introduction:**

Child development is a multidimensional process influenced by socioeconomic conditions. The Programa Criança Feliz (PCF), Brazil’s largest home-visiting initiative, uses the Formulário de Observação Inicial do Desenvolvimento Infantil (FOIDI) to monitor developmental milestones. Despite being widely implemented, the FOIDI had not previously undergone formal validation. This study evaluated configural and metric validity of FOIDI in a representative sample of Brazilian children aged 0–36 months.

**Methods:**

A total of 183 974 assessments from 82 797 children in PCF (2020–2023) were analysed. Cohorts for seven age groups were divided into three independent partitions. Initially, Horn’s parallel analysis determined the number of underlying factors. Confirmatory factor analyses followed, using weighted least-squares mean and variance adjusted estimator and polychoric correlation matrices. Model fit was evaluated using root mean square error of approximation, comparative fit index and Tucker–Lewis index. Items with factor loadings<0.40 were considered unreliable. Modification indices (MIs; univariate Lagrange multiplier) and expected parameter changes were used to identify potential cross-loadings and residual correlations. Convergent validity was estimated using average variance extracted (AVE≥0.50); discriminant validity was assessed by comparing the square root of AVE with factor correlations.

**Results:**

Modules exhibited unidimensional (M3–M7) or bidimensional structures (M1–M2), with factor loadings ranging from 0.519 to 0.903. AVE was≥0.50 for all factors evaluated, supporting convergent validity. Discriminant validity was confirmed within the bidimensional modules. The removal of redundant items increased model parsimony without compromising informativeness.

**Conclusion:**

The FOIDI demonstrated robust configural and metric validity, capturing core aspects of child development across different age groups in contexts of socioeconomic vulnerability. The refined instrument proved suitable for home-visiting programmes, offering a practical, reliable and scalable tool for developmental surveillance and the support of public policies.

**WHAT IS ALREADY KNOWN ON THIS TOPIC**

- Despite the wide availability of instruments for assessing child development, their internal structure is often not subjected to robust psychometric analyses.
- The *Formulário de Observação Inicial do Desenvolvimento Infantil* (FOIDI), used in Brazil’s *Programa Criança Feliz*, had not been formally validated.

**WHAT THIS STUDY ADDS**

- Provides the first national evidence of the configural and metric validity of the FOIDI, analysing more than 180 000 assessments of Brazilian children aged 0–36 months.
- Refines the FOIDI by identifying and removing redundant items, resulting in a parsimonious and psychometrically consistent version.

**HOW THIS STUDY MIGHT AFFECT RESEARCH, PRACTICE OR POLICY**

- Supports the use of the FOIDI as a reliable, low-cost tool for developmental surveillance in home-visiting programmes, particularly in vulnerable contexts.

## Introduction

 Child development (CD) refers to the behavioural, biological, physiological and psychological changes that occur from conception to maturity.^1 2^ It is widely recognised as a multidimensional phenomenon, encompassing a range of interdependent skills and abilities.^2^ Although disagreement exists in the literature with respect to the precise boundaries and measurements of these dimensions,^1 3^ key domains include language, motor skills, cognitive abilities, socioemotional functioning and self-regulation.^1 2^

In low-income and middle-income countries, it is estimated that 250 million children aged 5 years or less are at risk of not achieving full developmental potential.^4^ This vulnerable period is marked by rapid physical growth and significant neuroplasticity.^5^ Deficits in CD may result in permanent functional limitations, constraining educational attainment and economic opportunity throughout life.^6^ Evidence highlights numerous contextual, individual, biological and psychosocial risk factors associated with developmental delays, defined as the failure to achieve expected milestones in one or more developmental domains.^7–10^ Early detection of such delays is crucial for preventing lifelong disabilities,^6^ underscoring the importance of systematic developmental monitoring and evaluation.

Screening, monitoring and surveillance represent effective strategies for tracking a child’s developmental progress.^11^ Fernald *et al*^1^ identified 147 available tools for assessing CD in children aged 0–8 years. Despite this broad availability, the monitoring and evaluation of developmental outcomes in the context of routine health systems faces considerable challenges, including difficulties with outcome measurement itself.^3^

In Brazil, there is a persistent lack of information regarding how children are progressing towards reaching full development.^5^ This information gap persists due to a scarcity of standardised instruments, Brazil’s geographic and sociodemographic diversity, as well as the use of differing tools.^12^ The lack of population-level data places constraints on targeted initiatives and programmes aimed at mitigating developmental delays in vulnerable children.^13^ In this context, using standardised instruments in large-scale maternal, neonatal and child health programmes offers an opportunity to address these challenges.^3^

One such initiative is the *Programa Criança Feliz* (PCF, or ‘Happy Child Program’), launched in 2016 via governmental decree (no. 8869). PCF employs a home-visiting methodology^14^ to target pregnant women, children up to 3 years of age living in social vulnerability, and children up to age six who are registered in *Programa Bolsa Família,* a conditional cash transfer programme. Social workers strengthen caregiver–child bonding, stimulate CD, and promote effective parenting practices.^14^

A key feature of the programme is its capacity to monitor developmental gains through a specific, easy-to-administer instrument that enables the recording of everyday observations on a child’s progress and difficulties. This tool, the *Formulário de Observação Inicial do Desenvolvimento Infantil* (FOIDI; Initial Child Development Observation Form), tracks developmental milestones across different age groups.^15^ Although it has been widely used since PCF’s inception, the FOIDI has not yet been formally evaluated with respect to its reliability or validity.

The present study initiates the validation process by examining, across age groups, the instrument’s dimensional structure, the relevance and reliability of its items, and the FOIDI’s convergent factor validity (CFV) and discriminant factor validity (DFV).

## Methods

### Participants and sampling

The present study used anonymised, individual-level data for children aged 0–3 years. Data spanning the years 2020–2023 were obtained from the e-PCF System,^16^ curated by the Center for Data and Knowledge Integration for Health (CIDACS) at the Oswaldo Cruz Foundation (FIOCRUZ). All procedures performed by CIDACS involve strict technical and physical safeguards for data management, documentation, storage and protection, as required by the General Data Protection Law (LGPD) and Resolution No. 466/2012 of the National Research Ethics Commission of the National Health Council.^17 18^

**Figure 1:**
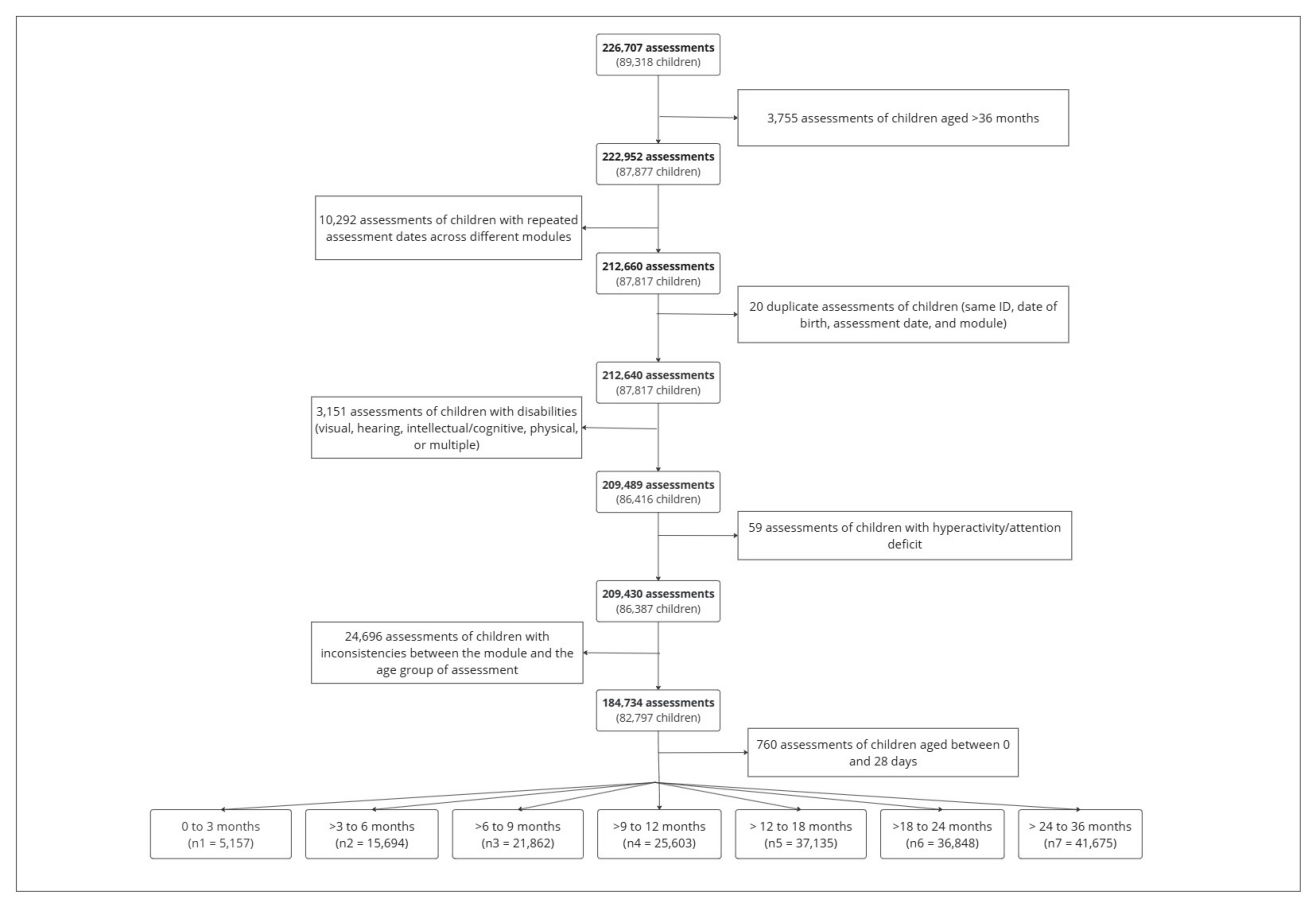
Flowchart detailing the construction of the database and the relevant exclusion criteria.

Given the PCF’s strategy of ongoing developmental monitoring, the database included 89 318 children, with the following distribution concerning the frequency of developmental assessments: 45.6% had one assessment, 21.8% two, 13.8% three, 8.0% four, 5.7% five, 3.5% six, 1.3% seven and 0.26% had eight assessments. Since 54.4% of the children were followed across more than one age group, this resulted in a total of 226 707 developmental milestone records. After applying ineligibility criteria and data cleaning, the final analytic sample consisted of 183 974 assessments, as shown in figure 1. This total corresponded to 82 797 children, distributed across seven age-defined strata: >0 to 3 months (*n*_1_=5157); >3 to 6 months (*n*_2_=15 694); >6 to 9 months (*n*_3_=21 862); >9 to 12 months (*n*_4_=25 603); >12 to 18 months (*n*_5_=37 135); >18 to 24 months (*n*_6_=36 846); and >24 to 36 months (*n*_7_=41 675).

### Data collection instrument and measurement procedures

The FOIDI was developed using four sources: the Initial Child Development Diagnostic Instrument (*Marco Zero*) from the *Primeira Infância Melhor* (PIM) Program^19^; the Child Development Monitoring Form^20^; the CD gains table from the Latin American Reference Center for Preschool Education (Cuba)^21^ and the Denver II Scale,^22^ originally developed in the USA. The instrument comprises 65 items distributed across seven modules (by age group), covering four domains of development: motor (control and coordination of gross limb movement and fine finger movements); cognitive (skills related to knowledge acquisition and processing); socio-affective (intentional behavioural and cognitive regulation) and language (capacity for comprehension and expression of verbal communication).^23^ The version of the instrument evaluated in this study is presented in online supplemental table S1. Each developmental milestone is assessed via three response options: ‘able to do’, ‘able to do with help’ or ‘not yet able to do’.

During scheduled, age-appropriate visits, home visitors asked or supervised each child evaluated in performing relevant milestones and then recorded their findings on a printed form. These observations, along with additional sociodemographic data regarding the child and his/her family, were later entered into the e-PCF system.^15^

### Statistical analysis

For all analyses, samples from the seven age groups were randomly divided into three equally sized, mutually exclusive partitions, yielding the following sample sizes for each stratum: *pn*_1_=1719, *pn_2_*=5231, *pn_3_*=7287, *pn_4_*=8534, *pn_5_*=12 378, *pn*_6_=12 283 and *pn_7_*=13 892. This partitioning generated independent subsamples, supporting a stepwise analytical protocol: the primary goal was to identify initial patterns, then to refine model evaluation, and finally to verify the replicability and consistency of the obtained results.

The analysis involved four sequential steps. Initially, dimensional structure was explored within the *p*_1_ partition for each age group by employing Horn’s parallel analysis (HPA) based on principal component analysis.^24^ Instead of the typical 1.0 cut-off reference for factor retention as suggested in the literature, a more sensitive threshold of 0.95 was adopted. Where more than one factor was suggested, exploratory structural equation models (ESEM) using geomin oblique rotation were applied before moving to the next step to clarify item-factor allocations.^25^ If HPA indicated a single factor, the analysis proceeded to the next step directly.

In the second phase, confirmatory factor analyses (CFA) were performed using *p_2_* partition samples to further scrutinise dimensional structure. During this exploratory phase, modification indices (MI; univariate Lagrange multiplier) and their corresponding expected parameter changes (EPC)^26 27^ were analysed to determine possible cross-loadings (in multidimensional models) and residual correlations (in all models). MI values quantify reductions in χ^2^ when a parameter is added, while EPC estimates the absolute parameter magnitude if freely estimated.^27^

Cross-loading was defined when at least two item loadings exceeded 0.30.^27^ With regard to residual correlations, only MI/EPC values pertaining to unusually high standardised correlations above 0.30 were initially examined. When such values were confirmed, those item pair residuals were then freely estimated. A final correlation value above 0.30 was interpreted as evidence of conditional independence violation^28^; in such instances, the item exhibiting the lower factor loading within the problematic pair was excluded. In this analysis, the magnitude of factor loadings was also used to assess empirical item reliability: loadings below 0.40 were considered unreliable and therefore of unsuitable discriminant capacity.^29^

The third step evaluated CFV using the *p_2_* partitions for each group. Average variance extracted (AVE), ranging from 0 to 1, was measured to quantify the degree of variance captured by a factor relative to the variance attributed to measurement error. CFV was confirmed where AVE≥0.50, indicating that explained variance outperformed measurement error.^27 30^

DFV was further assessed for multidimensional structures. Verification was based on factor correlations, with values ≥0.80 indicating possible violations.^27^ Additionally, statistical contrasts between factor correlation and AVE were evaluated by comparing, for each factor, the square root of AVE with the factor’s correlation value. Where DFV was determined, more information is expected to flow from each factor to its items than between the factors themselves.^31^ DFV for any factor *k* was rejected only when the difference $\sqrt{AVE_{\left(f_{k}\right)}\;}-\emptyset_{\left(f_{k},\;f_{\neq k}\right)}$ proved negative and statistically significant. In all other situations, DFV was corroborated. Only if factor correlations were ≥0.85 and significantly greater than the AVE test result was DFV considered to be rejected.^27 30^ A fourth and final step repeated these model validations in each of the seven age groups of the *p_3_* partition.

Given the trichotomous response structure of the items, factor analyses were conducted using the robust weighted least squares mean and variance adjusted (WLSMV) estimator and polychoric correlation matrices in Mplus V.8,^31^ which also handles missing data.^32^ The following three indices were used to evaluate model fit: root mean square error of approximation (RMSEA), which assesses the model’s approximation error per degree of freedom, allowing comparisons between models of different complexities (values≤0.06 indicate acceptable fit; values>0.10 indicate model rejection); comparative fit index (CFI) and Tucker–Lewis index (TLI) with values ≥0.95 considered acceptable and those <0.90 rejected—both of the latter indices range from 0 to 1, measuring incremental fit by comparing a specific model to a more restrictive (‘null’) model.^27^

## Results

Table 1 presents the distribution of sociodemographic characteristics within the studied sample. Most of the children evaluated were classified as black or brown (65.0%) and male (50.7%). The primary caregiver was predominantly the mother (97.1%), with incomplete or complete high school education (56.6%). Only 8.6% of caregivers reported employment outside the home.

**Table 1:** Sociodemographic characteristics of the participating children and their primary caregivers (2020–2023)

Variable	n	%
**Sex**		
Male	41 938	50.7
Female	40 839	49.3
Race/skin colour*		
White	21 534	26.0
Black	2760	3.3
Brown (*Parda*)	57 592	61.7
Yellow (Asian)	511	0.6
Indigenous	377	0.5
Primary caregiver†		
Mother	80 406	97.1
Father	428	0.5
Stepfather/stepmother	19	0.02
Brother/sister	94	0.1
Grandfather/grandmother	1511	1.8
Other	309	0.4
Primary caregiver education level		
Illiterate	871	1.0
Functionally illiterate	922	1.1
Incomplete/complete elementary school	30 000	36.2
Incomplete/complete high school	46 817	56.6
Incomplete/complete higher education	4155	5.0
Primary caregiver employment		
Yes	7113	8.6
No	75 651	91.4
Age by module (in months)‡	**Mean**	**±SD**
M1	2.4	0.3
M2	4.7	1.0
M3	7.4	1.1
M4	10.4	1.1
M5	14.5	2.2
M6	20.4	2.2
M7	27.8	3.7

Total sample comprised 82 797 children. Reported frequencies may not correspond to the full sample size due to missing data in some variable categories.

* Self-reported classification according to the Brazilian Institute of Geography and Statistics categories.

† 'Other’ category includes uncle/aunt, sibling, neighbour or other caregivers identified as responsible for the child at the time of data collection.

‡ Modules (M1–M7) correspond to the standardised age groups monitored by the programme, in months.

In the first analytic stage, HPA indicated a bifactorial structure in modules 1 (*eig_M1(f1)_*=5.313; *eig_M1(f2)_*=1.064) and 2 (*eig_M2(f1)_*=5.401; *eig_M2(f2)_*=1.179). ESEM subsequently specified item-factor assignments: in module 1, the first factor included items i1, i2, i3, i7, i8 and i9, while the second comprised items i4, i5 and i6. For module 2, one factor encompassed i1, i2, i6, i7 and i8, and the other consisted of i3, i4, i5 and i9. Modules 3–7 exhibited a unifactorial structure (*eig_M3(f1)_*=4.765; *eig_M4(f1)_*=4.124; *eig_M5(f1)_*=3.692; *eig_M6(f1)_*=5.384; *eig_M7(f1)_*=7.098).

In the second stage of analysis, CFA performed using partition *p_2_* did not reveal significant cross-loadings in either bifactorial module (M1 or M2). In all modules, factor loadings for all items exceeded the established threshold (λ≥0.40). As shown in table 2, certain modules exhibited MI and EPC suggestive of residual item correlations. Upon freely estimating these parameters, items with lower factor loadings within correlated pairs were excluded.

**Table 2:** Modification indices, expected parameter change and freely estimated residual correlations of the respecified models by module

Modules	Items	Modification indices	Expected parameter change	Residual correlation
M3	i1↔i2	383.470	0.395	0.348
M5	i1↔i2	906.225	0.935	0.549
M6	i4↔i5	170.530	0.277	0.372
	i4↔i6	166.430	0.263	0.360
	i5↔i6	426.180	0.437	0.453
M7	i6↔i7	526.168	0.448	0.378
	i11↔i12	482.679	0.471	0.412

In modules 3 and 5, residual correlations between items i1 and i2 led to the exclusion of i1 in both modules. Within module 6, three item pairs (i4↔i5, i4↔i6, i5↔i6) presented high MI/EPC values suggestive of residual correlations, and removing any two resolved the violation of conditional independence. The retained item in each case (i5) was selected based on relatively higher factor loading. Similarly, in module 7, correlation between i6↔i7 and i11↔i12 favoured the retention of i7 and i11. No residual correlations were detected in modules M1, M2 or M4. Further details on the estimated loadings and residual correlations for all models are available as online supplemental tables S2 to S8.

In stage 3 of the analysis, all modules analysed in partition *p_2_* showed AVE values greater than 0.50. For module 1, the difference between the √AVE of factors 1 (0.830) and 2 (0.781) and their correlation were positive (0.088 and 0.039; p<0.05). In module 2, the √AVE of the first factor (0.858) also exceeded the factor correlation, resulting in a positive difference (0.069; p<0.01). In contrast, for the second factor, √AVE (0.720) was lower than the factor correlation, yielding a negative difference (–0.069; p<0.01).

The final stage retested all models on partition *p_3_*. Confirmatory results, as presented in table 3, demonstrated strong fit indices: factor loadings ranged from 0.519 to 0.903, and all AVE values remained above 0.50. As previously observed in partition *p_2_*, differences between √AVE and correlations remained positive for both factors in module 1 (0.818 and 0.788; p<0.01) and for the first factor in module 2 (0.075; p<0.001), while a negative result was obtained for the second factor in module 2 (−0.076; p<0.05).

**Table 3:** Confirmatory factor analysis of the Initial Child Development Observation Form used in the Criança Feliz Program (partition 3; *p_3_*)

Item	Module 1		Module 2		Module 3	Module 4	Module 5	Module 6	Module 7
	*λ_i_*_(f1)_ *	*λ_i_*_(f2)_ *	*λ_i_*_(f1)_ *	*λ_i_*_(f2)_ *	*λ_i_*_(f1)_ *	*λ_i_*_(f1)_ *	*λ_i_*_(f1)_ *	*λ_i_*_(f1)_ *	*λ_i_*_(f1)_ *
i1	0.721	–	0.813	–	–	0.641	–	0.772	0.827
i2	0.819	–	0.885	–	0.654	0.769	0.733	0.758	0.703
i3	0.722	–	–	0.702	0.731	0.736	0.755	0.735	0.807
i4	–	0.820	–	0.839	0.698	0.685	0.652	–	0.830
i5	–	0.889	–	0.519	0.754	0.759	0.751	0.642	0.715
i6	–	0.667	0.892	–	0.740	0.705	0.780	–	–
i7	0.897	–	0.903	–	0.691	0.781	–	0.857	0.715
i8	0.869	–	0.793	–	0.868	–	–	0.746	0.840
i9	0.862	–	–	0.734	0.798	–	–	0.726	0.834
i10	–	–	–	–	–	–	–	–	0.666
i11	–	–	–	–	–	–	–	–	0.722
i12	–	–	–	–	–	–	–	–	–
									
AVE	0.669	0.622	0.737	0.501	0.554	0.528	0.541	0.563	0.590
Φƒ(1)↔ƒ(2)†	0.708		0.784		–	–	–	–	–
RMSEA	0.042 (0.034 to 0.050)		0.061 (0.057 to 0.066)		0.057 (0.053 to 0.062)	0.051 (0.047 to 0.056)	0.033 (0.027 to 0.040)	0.052 (0.048 to 0.056)	0.03 (0.033 to 0.037)
CFI	0.992		0.978		0.980	0.986	0.997	0.982	0.984
TLI	0.989		0.970		0.972	0.979	0.993	0.973	0.979

* Loadings.

† Factor correlation.

AVE, average variance extracted; CFI, comparative fit index; i, item; RMSEA, root mean square error of approximation and 95% CI; TLI, Tucker–Lewis index .

## Discussion

The FOIDI was found to demonstrate factorial and metric validity, further establishing its utility as a screening and monitoring tool for the evaluation of CD within the scope of the PCF. The factorial structure observed herein suggests the instrument’s psychometric appropriateness for population-level application in home visitations.

Although the design of the FOIDI was based on a theoretical model consisting of four domains, communication and language, socioemotional, motor and cognitive,^15^ the present results indicate a distinct pattern. As revealed in our findings, the modules showed essentially bidimensional structures in the first two age groups (M1 and M2), compared with unidimensionality in the later age groups (M3 to M7). Across all seven modules, the factorial configurations were well-delineated, factor loadings exceeded the established reliability threshold (λ≥0.40), and AVE values surpassed established cut-off minimums (≥0.50), supporting CFV consistent with contemporary psychometric standards.^27 29 33^

CD is consistently recognised in the literature as a multidimensional process interconnecting various skills and abilities.^2^ The factorial structure of the FOIDI, however, highlights ongoing methodological challenges in operationalising multidimensionality through psychometrically validated instruments. The literature points precisely to this discrepancy between theoretical conception and empirical evidence. Although multiple domains are postulated, there is divergence in the literature regarding which domains these are, and few instruments can validly and consistently represent such a structure.^3 34^

One factor contributing to this challenge is the lack of systematic structure validation in many CD assessment instruments.^34^ The influential Denver II, from which the FOIDI partly draws, is traditionally described as representing four developmental domains: personal-social, fine motor-adaptive, language and gross motor.^22^ Yet as a review by Silva *et al*^34^ argued, no factorial analysis has robustly substantiated its multidimensional structure. It is important to note that the Denver II items included in the FOIDI were mainly selected based on clinical expertise and expert judgement rather than rigorous psychometric or content validity analyses.^34^ Moreover, reducing the item pool from 125 (Denver II) to 65 on the FOIDI likely increased statistical parsimony, but may ultimately restrict the estimative capability of the original domains.

It is also notable that the socio-affective dimension was absent from modules M1 and M5, being represented by only one item each in M2, M3 and M6 (*“recognizes familiar people and cries in front of strangers,” “pays attention when hearing their name” and “eats using a utensil with their own hand”,* respectively). While the importance of socioemotional assessment for early detection of neurodevelopmental risks is well-established, measurement of this dimension through milestone-based frameworks suffers limitations; relatively scarce standardised indicators tend to emphasise self-help and adaptive skills rather than the subtleties of social engagement or emotional regulation. This limitation applies not only to the FOIDI but is also relevant in other approaches to developmental surveillance.^35 36^

The present results indicate that, considering the current set of items, unidimensional or bidimensional modelling are a more accurate fit for the FOIDI compared with the initially postulated four-dimensional structure. This finding reflects not only the limitations of this particular screening instrument, but also the general challenge of translating multidimensional theories on CD into consistent and reliable psychometric tools for evaluation purposes.

In modules M1 and M2, which both revealed bidimensionality, the motor domain was distinctly differentiated from other constructs. In M1, items 4 (*“holds head steady when lifted”*)*,* 5 (*“when placed on the stomach, momentarily lifts the head and part of the torso”*) and 6 (*“grasps objects placed within reach”*)*,* which all originally categorised as motor milestones,^16^ clustered together as a single factor in the present analysis. An analogous separation was found in M2, where items 3 (*“moves from prone to supine position and vice versa”*)*,* 4 (*“grasps toys and holds them for some time”*) and 5 (*“sits unsupported for a period of time”*) formed a distinct motor component. This pattern highlights the developmental prominence of the motor domain, aligning with established theoretical and normative data regarding the rapid and observable progression of motor skills, particularly during early infancy.^2 37^

The second factor in these two modules comprised a heterogeneous mix of socio-affective, communicative and attention-related items. Notable socio-affective items in M1 were *“shows pleasure and discomfort”* and *“smiles in response to a person’s face,”* while *“recognizes familiar people and cries in front of strangers”* and *“babbles and smiles during interaction with others”* were important in M2. Communicative milestones in M1 included *“produces sounds as a form of communication”* and *“recognizes and responds to the mother/caregiver’s voice,”* compared with M2: *“recognizes the voice of certain people”* and *“varies the volume of vocalizations.”* Early social attention markers in M1 comprised *“fixes gaze for a few seconds on people’s faces or objects”* and *“tracks moving people or objects with gaze”* versus “*searches for objects in front of them with the eyes*” in M2. Collectively, this configuration constitutes a socio-affective-communicative construct that plausibly reflects the integration of interactive, responsive and communicative behaviours in early infancy prior to becoming fully differentiated into specific subdomains.^36^

For modules M3–M7, the unidimensional factor structure demonstrated consistency even after statistical adjustments for residual item correlations. While these models comprised internally consistent sets of items and reflected parsimoniousness, the identification of items with high residual correlation between pairs suggested violations of local independence and, therefore, the need for further refinement.^26 27^

The interpretation of these statistical findings is crucial for guiding measurement and intervention.^27^ In module M3, a high residual correlation was observed between gross motor milestones *“begins to crawl and/or creep”* (i1) and *“sits and maintains balance”* (i2), both both reflecting postural control. Likewise, within module M5, a residual correlation was observed between *“walks with balance”* (i1) and *“kicks a ball”* (i2), which both evaluate aspects of axial stability. Substantive analysis suggested that these pairs measure foundational, overlapping aspects of motor development, resulting in content redundancy.^38^

For language and cognitive domains in modules M6 and M7, residual correlations appeared to result from similar skill clusters. In M6, items focused on expressive language capacity, such as *“speaks three-word phrases”* (i4), *“names some everyday objects”* (i5), and *“begins to use pronouns, eg, my, your”* (i6), were notable. In M7, skills associated with *“select similar objects by color and shape”* (i6) and *“build towers or bridges with more than three elements”* (i7), as well as *“says goodbye when leaving a place”* (i11) and *“accepts interaction with other people, even if unfamiliar”* (i12) suggested grouping by set formation ability and social competence, respectively.

The post hoc exclusion of items with lower factor loadings yielded refined factor structures and enhanced construct validity. The procedure to exclude items due to redundancy was executed with methodological caution, relying on both statistical and substantive criteria. Such adjustments, endorsed by psychometric literature, facilitated the attainment of more parsimonious models and reduced bias without sacrificing informativeness.^27 38^

We also observed consistent evidence of CFV in all modules, as indicated by AVE values exceeding the prespecified threshold (≥0.50). This finding demonstrates that each factor’s items collectively account for more variance in the underlying construct than measurement error, reinforcing the interpretability and robustness of the FOIDI scales.^33^ Additionally, DFV was confirmed for the two bidimensional modules (M1 and M2). In these modules, formal testing of the difference between the square root of AVE and the factor correlations yielded statistically significant positive values, with the exception of the second factor of M2, for which a significant negative difference emerged.^32^ Notwithstanding this exception, factor intercorrelations for both modules remained below the accepted threshold of 0.80, suggesting that the constructs are empirically separable and conceptually distinct.^27^ It follows that the subconstructs derived from the FOIDI are capable of explaining more variance through their own items than from shared variance with other factors, thus meeting contemporary psychometric expectations.^33^

An important strength of this study lies in the use of a large, nationally representative sample of approximately 80 000 children from diverse Brazilian regions, which facilitates the generalisability and applicability of the findings from the present analysis. Additional robustness was imparted through the use of a cohesive analytic strategy^28–33^ that integrated contemporary psychometric criteria for validation with the production of reliable data for scale refinement. The deliberate partitioning of the sample into independent subsamples enabled rigorous cross-validation, allowing the stability and replicability of the factorial solutions and validation indices to be systematically evaluated across varying segments of the studied population.

Nonetheless, certain limitations warrant discussion. One is the secondary nature of the dataset, which was generated by field-based assessments carried out by PCF home visitors. This introduces the possibility of measurement bias stemming from subjective evaluator judgement, variability in the implementation of the protocol, and inconsistencies in response recording or activity administration. If such bias were present, it could have impacted the psychometric properties estimations and, by extension, the accuracy of substantive interpretations regarding developmental outcomes.^27 39^ Even so, the overall satisfactory factor structures and reliability indices observed herein point to the validity of the conclusions drawn from the analyses.

Another noteworthy limitation concerns the exclusion of approximately 24 000 assessments that fell outside the appropriate age range for the modules applied, as depicted in the study flowchart (figure 1). The loss of roughly 10% of the dataset could, in theory, compromise our study’s findings if the exclusions reflected a systematic (non-random) bias.^38^ However, we submit that the excluded cases most likely reflect clerical or procedural errors unrelated to the children’s actual developmental status, rendering it improbable that the central findings or inferences were substantially compromised.

In summary, the present study provides robust evidence that the FOIDI is well-suited to accurately capture developmental constructs across a broad age range, even in the context of an abbreviated item set. The refined instrument retains high reliability, precision and non-redundancy of items, successfully providing convergent factorial validity across all modules and discriminant validity in the two that exhibited bidimensionality. The methodological refinement employed—anchored by the systematic elimination of redundant items—yielded a streamlined version that is both more parsimonious and efficient, facilitating application in large-scale monitoring and home-visiting contexts without sacrificing scientific rigour.

The implications of the present findings are twofold: the FOIDI can be broadly recommended for developmental surveillance in diverse populations, and its ongoing application will further inform and verify its structural validity in a variety of real-world settings, including contexts lacking active social intervention. Moving forward, broader studies should attempt to enhance the instrument’s scale validity^33^ across each age band; namely, whether items within each module can be considered hierarchically and cumulatively ordered (scalability), which would reflect a progressive increase in the intensity of latent traits.^40^ This type of analysis, which remains rare in the international literature focused on validation studies, could help clarify whether FOIDI modules operate as true scales, ultimately enhancing their value in classifying and ranking children’s developmental levels while also identifying the most vulnerable who would benefit from targeted intervention.

## Conclusion

In conclusion, the present study demonstrates that the FOIDI is a valid and reliable psychometric instrument for assessing core aspects of CD across multiple age groups, even in its newly streamlined and more parsimonious form. The refined instrument maintains strong convergent validity across all modules and discriminant validity for those with multidimensional structure, while eliminating redundancy and improving applicability for large-scale monitoring within home-visiting and public health contexts. By supporting systematic developmental surveillance, especially among populations facing socioeconomic vulnerability, the FOIDI can contribute to more effective policy and intervention, directly aligning with the objectives of the Sustainable Development Goals to ensure healthy development and reduce inequities in early childhood. Further research should deepen the investigation of scale validity and scalability within each module, which may help clarify the extent to which the FOIDI can fulfil its potential for classification and risk stratification for developmental delay.

## Supplementary material

10.1136/bmjpo-2025-004362 online supplemental file 1

## Data Availability

Data are available upon reasonable request.
