# Supplementary material for: Psychometric evidence for assessing early childhood development in Brazil: the Child Development Diagnostic Form in the Criança Feliz Program
Source: BMJ Paediatr Open. 2026 Aug 4;10(1):e004362. doi: 10.1136/bmjpo-2025-004362 (PMC13448607; doi:10.1136/bmjpo-2025-004362)
Supplement: Supplementary file 1 [file bmjpo-2025-004362.reviewer_comments.pdf]

PEER REVIEW HISTORY

BMJ Paediatrics Open publishes all reviews undertaken for accepted manuscripts. Reviewers are asked to complete a checklist review form and are provided with free text boxes to elaborate on their assessment. These free text comments are reproduced below.

ARTICLE DETAILS

**TITLE (PROVISIONAL)**	PSYCHOMETRIC EVIDENCE FOR ASSESSING EARLY CHILDHOOD DEVELOPMENT IN BRAZIL: THE CHILD DEVELOPMENT DIAGNOSTIC FORM IN THE CRIANÇA FELIZ PROGRAM
**AUTHORS**	Cerqueira, Ellayne<br>Reichenheim, Michael<br>Rodrigues Alves, Ana Angélica<br>Rodrigues Dutra, Ricardo<br>Leite de Moraes, Claudia<br>Costa, Priscila Ribas de Farias<br>Silva, Rita de Cássia<br>L. Barreto, Mauricio

VERSION 1 – REVIEW

**REVIEWER NAME**	Gowda, Vykuntaraju
**REVIEWER AFFILIATION**	Indira Gandhi Institute of Child Health, Pediatric Neurology
**REVIEWER CONFLICT OF INTEREST**	No Conflict of Interest
**AI DISCLOSURE**	No, I have not used AI
**DATE REVIEW RETURNED**	26-Jan-2026

**GENERAL COMMENTS**	Very well written article.<br>Any correlation done with respect etiology for developmental delay

**REVIEWER NAME**	Sehgal, Dr Rachna
**REVIEWER AFFILIATION**	VMMC and SJH, Pediatrics
**REVIEWER CONFLICT OF INTEREST**	No Conflict of Interest
**AI DISCLOSURE**	No, I have not used AI
**DATE REVIEW RETURNED**	02-Mar-2026

**GENERAL COMMENTS**	Congratulations for a huge work involving a large number of patients and assessments. Following responses are desired.<br>1. Abbreviations in the abstract need to be expanded.<br>2. Was any gold standard used to compare psychometric properties of Child Development Diagnostic form?<br>3. The name of the form itself is misleading as this instrument has been designed for surveillance and not diagnosis.<br>4. Which items had a factor loading of <0.5, which were later dropped from the instrument as mentioned in the methods?

VERSION 1 – AUTHOR RESPONSE

Reviewer: 1
Dr. Vykuntaraju Gowda, Indira Gandhi Institute of Child Health, PROF AND HOD OF PEDIATRIC NEUROLOGY, IGICH
Comments to the Author
Very well written article.

1. Any correlation done with respect etiology for developmental delay?
We refrained from assessing the relationship between what the instrument measures and the etiology of developmental delay, as we believe that this falls outside the main scope of the present article. This choice was guided by the methodological framework proposed by Reichenheim and Bastos (2021), according to which the evaluation of the development process of a measurement instrument involves distinct and sequential stages.
Following an initial phase aimed at obtaining a prototype (essentially based on a qualitative research approach), the instrument is then examined in a second phase through successive psychometric evaluations, which may themselves be divided into two major stages. In the first, the internal structure of the prototype instrument is assessed, addressing its configural, metric, and scaling properties. In the second stage, the focus shifts to the systematic scrutiny of the results derived from the use of the instrument (e.g., scores from its component scales), examining whether these align with theoretical expectations in relation to other constructs or clinical criteria, including specific etiologies.
Our current study focused strictly on the first aspect, namely the internal structure, in order to ensure the psychometric robustness of the Child Development Diagnostic Form (FDDI). We consider the establishment of these properties to be a prerequisite for proceeding to what may be regarded as external validation (Reichenheim & Bastos, 2021).
We do agree that investigating associations with theoretically relevant external variables is a crucial step in the overall evaluation of an assessment tool, a point we explicitly highlighted in our recommendations for future research in the Discussion section.
We hope that our approach is acceptable.

Reviewer: 2
Dr. Dr Rachna Sehgal, VMMC and SJH
Comments to the Author
Congratulations for a huge work involving a large number of patients and assessments. Following responses are desired.

1. Abbreviations in the abstract need to be expanded.
All abbreviations in the abstract, including the psychometric indices (RMSEA, CFI, TLI, and WLSMV) and validity measures (AVE), have now been expanded upon their first mention.

2. Was any gold standard used to compare psychometric properties of Child Development Diagnostic form?

We would like to clarify that the use of a reference measure presupposes an external validation approach (such as concurrent validity). As previously indicated in our response to Reviewer 1, this specific type of validation is outside the scope of the current study.
We reiterate that this endeavor is by no means to be disregarded; on the contrary, it is an essential part of a future research agenda, as we have explicitly stated in the final section of the Discussion.
Furthermore, due to the pragmatic nature of the health surveillance program from which these data originate, the collection of information from a reference instrument was not foreseen in the original design. An initiative in this direction would necessarily require a prospective study, perhaps involving smaller and controlled samples using face-to-face interviews to compare the FDDI with an established international reference measure. This initial validation of the internal structure (configural and metric) provides the indispensable psychometric foundation for such future head-to-head clinical

comparisons.

3. The name of the form itself is misleading as this instrument has been designed for surveillance and not diagnosis.
We fully agree with the reviewer's observation. The term "diagnostic" may be misleading, as the instrument is intended for developmental surveillance and monitoring within the scope of a social program, rather than providing a clinical diagnosis. We have thus replaced "Child Development Diagnostic Form" (FDDI) with "Initial Child Development Observation Form" (Formulário de Observação Inicial do Desenvolvimento Infantil) throughout the manuscript. This nomenclature is more accurate and in line with the terminology used in the official Family Visit Guide of the Programa Criança Feliz (Brasil, 2019). We believe this change clarifies the tool's purpose as a screening and surveillance instrument.

4. Which items had a factor loading of <0.5, which were later dropped from the instrument as mentioned in the methods?

We clarify that no item was excluded for having a factor loading below 0.50. Across all modules, the full set of items exhibited robust factor loadings, well above the established cutoff. As detailed in this section and shown in the Supplementary Tables (S2 to S8), all factor loadings in Partition 1 (in which no exclusions had yet been made) were high.
The exclusions mentioned in the Results section were based strictly on high residual correlations, which indicate a violation of the assumption of local independence (Brown, 2015; Reeve et al., 2007). The decision to remove specific items was made only during the model refinement phase (Partition 2 onward), with the aim of achieving greater parsimony and better fit:
• In Modules 3 and 5, item i1 was excluded because of its residual correlation with item i2.
• In Module 6, items i4 and i6 were removed to resolve residual correlations, while item i5 was retained.
• In Module 7, items i6 and i12 were removed because of correlations with items i7 and i11.
In each case, we retained the item from the residually correlated pair with the higher factor loading, although all items excluded on this basis had loadings above 0.50.
We have updated the manuscript to ensure this distinction is clear. Specifically, we added a descriptive statement to the Results section (Page 8, Paragraph 2) reporting that all items presented loadings above the 0.40 threshold. Furthermore, we have highlighted the original justification for exclusions—based solely on residual correlations—which was already present in the Results (Page 8, Paragraphs 2 and 3) and Discussion (Page 9, Paragraph 3) sections, ensuring these points are now easily identifiable.

References

Brasil. Ministério da Cidadania. (2019). Guia para visita domiciliar: manual / Ministério da Cidadania (1 ed. rev. atual.).
Brown, T. A. (2015). Confirmatory Factor Analysis for Applied Research (The Guilford Press, Ed.; second). www.guilford.com/MSS
Reeve, B. B., Hays, R. D., Bjorner, J. B., Cook, K. F., Crane, P. K., Teresi, J. A., Thissen, D., Revicki, D. A., Weiss, D. J., Hambleton, R. K., Liu, H., Gershon, R., Reise, S. P., Lai, J.-S., & Cella, D. (2007). Psychometric Evaluation and Calibration of Health-Related Quality of Life Item Banks Plans for the Patient-Reported Outcomes Measurement Information System (PROMIS).
Reichenheim, M., & Bastos, J. L. (2021). What, what for and how? Developing measurement instruments in epidemiology. Revista de Saude Publica, 55, 1–17. https://doi.org/10.11606/s1518-8787.2021055002813

VERSION 2 – REVIEW

**REVIEWER NAME**	Gowda, Vykuntaraju
**REVIEWER AFFILIATION**	Indira Gandhi Institute of Child Health, Pediatric Neurology
**REVIEWER CONFLICT OF INTEREST**	No Conflict of Interest
**AI DISCLOSURE**	No, I have not used AI
**DATE REVIEW RETURNED**	05-Jun-2026

**GENERAL COMMENTS**	Answered all queries appropriately.

**REVIEWER NAME**	Sehgal, Dr Rachna
**REVIEWER AFFILIATION**	VMMC and SJH, Pediatrics
**REVIEWER CONFLICT OF INTEREST**	No Conflict of Interest
**AI DISCLOSURE**	No, I have not used AI
**DATE REVIEW RETURNED**	10-Jul-2026

**GENERAL COMMENTS**	Acceptable responses to the initial observations.
