# Supplementary material for: Psychometric evidence for assessing early childhood development in Brazil: the Child Development Diagnostic Form in the Criança Feliz Program
Source: BMJ Paediatr Open. 2026 Aug 4;10(1):e004362. doi: 10.1136/bmjpo-2025-004362 (PMC13448607; doi:10.1136/bmjpo-2025-004362)
Supplement: Supplementary file 2 [file bmjpo-10-1-s001.docx]

Supplementary Table 1. The Initial Child Development Observation Form used by the Criança Feliz Program

|  | **Item** | **Developmental Milestones** | **Dimensions** | **Response** | | |
| --- | --- | --- | --- | --- | --- | --- |
|  |  |  |  | Can do alone | Can do with help | Cannot do yet |
| **Module 0** |  |  |  |  | | |
|  | i1 | Moro reflex: Opens and closes arms in response to stimulation | - |  | | |
|  | i2 | Posture: lying on back, legs and arms flexed, head turned to the side | - |  | | |
|  |  |  |  |  | | |
| **Module 1** | i1 | Shows signs of pleasure and discomfort? | Communication/Language |  | | |
|  | i2 | Smiles at a person’s face? | Socio-affective |  | | |
|  | i3 | Produces sounds as a form of communication? | Communication/Language |  | | |
|  | i4 | Holds head steady when lifted? | Motor |  | | |
|  | i5 | When placed face down, lifts head and part of the trunk momentarily? | Motor |  | | |
|  | i6 | Casually grasps objects placed within reach? | Motor |  | | |
|  | i7 | Fixates gaze for a few seconds on people’s faces or on objects? | Cognitive |  | | |
|  | i8 | Follows moving people or objects with eyes? | Cognitive |  | | |
|  | i9 | Recognizes and reacts to the voice of mother/caregiver? | Cognitive |  | | |
|  |  |  |  |  | | |
| **Module 2** | i1 | Recognizes close people and cries in front of strangers? | Socio-affective |  | | |
|  | i2 | Babbles and smiles during interaction with others? | Communication/Language |  | | |
|  | i3 | Turns from stomach to back and vice versa? | Motor |  | | |
|  | i4 | Grasps toys and holds them for some time? | Motor |  | | |
|  | i5 | Sits without support for some time? | Motor |  | | |
|  | i6 | Recognizes the voice of some people? | Cognitive |  | | |
|  | i7 | Looks for objects in front of them with eyes? | Cognitive |  | | |
|  | i8 | Varies the volume of vocalizations? | Communication/Language |  | | |
|  | i9 | Holds and transfers objects from one hand to the other? | Socio-affective |  | | |
|  |  |  |  |  | | |
| **Module 3** | i1 | Starts crawling and/or creeping? | Motor |  | | |
|  | i2 | Sits and maintains balance? | Motor |  | | |
|  | i3 | Grasps small objects with two fingers? | Motor |  | | |
|  | i4 | Places and removes objects of different sizes into/from a wide-mouthed container? | Cognitive |  | | |
|  | i5 | Looks for objects of interest when someone hides them on purpose? | Cognitive |  | | |
|  | i6 | Plays by throwing and retrieving objects? | Cognitive |  | | |
|  | i7 | Produces sounds and imitates others they hear? | Communication/Language |  | | |
|  | i8 | Pays attention when hearing their own name? | Socio-affective |  | | |
|  | i9 | Holds and transfers objects from one hand to the other? | Motor |  | | |
|  |  |  |  |  | | |
| **Module 4** | i1 | Takes small steps with support? | Motor |  | | |
|  | i2 | Handles, throws, and picks up toys? | Motor |  | | |
|  | i3 | Can do simple things, such as rocking a doll or walking a toy animal? | Socio-affective |  | | |
|  | i4 | Puts lids on and off round boxes? | Cognitive |  | | |
|  | i5 | Follows simple commands, such as “get the toy” or “give it to me”? | Cognitive |  | | |
|  | i6 | Uses at least one meaningful word? | Communication/Language |  | | |
|  | i7 | Makes hand and head gestures (no, bye-bye, claps hands)? | Socio-affective |  | | |
|  |  |  |  |  | | |
| **Module 5** | i1 | Walks with balance? | Motor |  | | |
|  | i2 | Kicks a ball? | Motor |  | | |
|  | i3 | Puts lids on and off boxes? | Cognitive |  | | |
|  | i4 | Combines at least two words? | Communication/Language |  | | |
|  | i5 | Drinks holding cup with own hand? | Motor |  | | |
|  | i6 | Builds a tower with two elements? | Cognitive |  | | |
|  |  |  |  |  | | |
| **Module 6** | i1 | Goes up and down low steps? | Motor |  | | |
|  | i2 | Builds a tower with at least three elements? | Cognitive |  | | |
|  | i3 | Puts lids on and off screw-top containers? | Cognitive |  | | |
|  | i4 | Says phrases with three words? | Communication/Language |  | | |
|  | i5 | Names some everyday objects? | Communication/Language |  | | |
|  | i6 | Starts using pronouns (e.g., mine, yours)? | Communication/Language |  | | |
|  | i7 | Holds a toy while walking? | Motor |  | | |
|  | i8 | Eats holding utensils with own hand? | Socio-affective |  | | |
|  | i9 | Follows up to three simple commands simultaneously? | Motora |  | | |
|  |  |  |  |  | | |
| **Module 7** | i1 | Understands most of what is heard? | Communication/Language |  | | |
|  | i2 | Says phrases with four or more words? | Communication/Language |  | | |
|  | i3 | Imitates simple adult behaviors? | Socio-affective |  | | |
|  | i4 | Runs safely? | Motor |  | | |
|  | i5 | Jumps with both feet together and/or stands on one foot? | Motor |  | | |
|  | i6 | Matches objects by color and shape? | Cognitive |  | | |
|  | i7 | Builds towers or bridges with more than three elements? | Cognitive |  | | |
|  | i8 | Scribbles and draws lines on paper? | Motor |  | | |
|  | i9 | Holds cup and spoon firmly? | Motor |  | | |
|  | i10 | Signals need to urinate or defecate? | Socio-affective |  | | |
|  | i11 | Says goodbye when leaving a place? | Socio-affective |  | | |
|  | i12 | Accepts interaction with other people, even strangers? | Socio-affective |  | | |

**Supplementary tables**

Supplementary Table 2. Confirmatory Factor Analysis of Module 01 of the Initial Child Development Observation Form from the Criança Feliz Program (partition 2).

| **Item** | **Factor 1** |  | **Factor 2** |
| --- | --- | --- | --- |
|  | ***λ_i_*_(f1)_ *^a^*** |  | ***λ_i_*_(f2)_ *^a^*** |
| i1 | 0.781 |  | - |
| i2 | 0.816 |  | - |
| i3 | 0.735 |  | - |
| i4 | - |  | 0.799 |
| i5 | - |  | 0.848 |
| i6 | - |  | 0.687 |
| i7 | 0.902 |  | - |
| i8 | 0.860 |  | - |
| i9 | 0.876 |  | - |
|  |  |  |  |
| $\emptyset_{f\left( 1 \right)\leftrightarrow f\left( 2 \right)}$ *^b^* | 0.742 (0.710; 0.774) | | |
|  |  | | |
| AVE | 0.690 |  | 0.610 |
|  |  |  |  |
| RMSEA *^c^* | 0.048 (0.040; 0.057) | | |
| CFI *^d^* | 0.990 | | |
| TLI *^e^* | 0.986 | | |
| *^a^* factor loading; *^b^* factor correlation; *^c^* Root-Mean-Square Error of Approximation (RMSEA) (90% Confidence Interval); *^d^* Comparative Fit Index (CFI)); *^e^* Tucker-Lewis Index (TLI). | | | |

Supplementary Table 3. Confirmatory Factor Analysis of Module 02 of the Initial Child Development Observation Form from the Criança Feliz Program (partition 2).

| **Item** | **Factor 1** |  | **Factor 2** |
| --- | --- | --- | --- |
|  | ***λ_i_*_(f1)_ *^a^*** |  | ***λ_i_*_(f2)_ *^a^*** |
| i1 | 0.830 |  | - |
| i2 | 0.881 |  | - |
| i3 | - |  | 0.721 |
| i4 | - |  | 0.828 |
| i5 | - |  | 0.556 |
| i6 | 0.894 |  | - |
| i7 | 0.888 |  | - |
| i8 | 0.795 |  | - |
| i9 | - |  | 0.747 |
|  |  |  |  |
| $\emptyset_{f\left( 1 \right)\leftrightarrow f\left( 2 \right)}$*^b^* | 0.789 (0.769; 0.809) | | |
|  |  | | |
| AVE | 0.737 |  | 0.518 |
|  |  |  |  |
| RMSEA *^c^* | 0.044 (0.040; 0.049) | | |
| CFI *^d^* | 0.998 | | |
| TLI *^e^* | 0.984 | | |
| *^a^* factor loading; *^b^* factor correlation; *^c^* Root-Mean-Square Error of Approximation (RMSEA) (90% Confidence Interval); *^d^* Comparative Fit Index (CFI)); *^e^* Tucker-Lewis Index (TLI). | | | |

Supplementary Table 4. Confirmatory Factor Analysis of Module 03 of the Initial Child Development Observation Form from the Criança Feliz Program (partition 2).

| **Item** | **Model 1** *^a^* | **Model 2 *^b^*** |
| --- | --- | --- |
|  | ***λ_i_*_(f1)_ *^c^*** | ***λ_i_*_(f1)_ *^c^*** |
| i1 | 0.604 | - |
| i2 | 0.652 | 0.652 |
| i3 | 0.741 | 0.740 |
| i4 | 0.682 | 0.683 |
| i5 | 0.750 | 0.756 |
| i6 | 0.738 | 0.728 |
| i7 | 0.687 | 0.692 |
| i8 | 0.864 | 0.864 |
| i9 | 0.806 | 0.807 |
|  |  |  |
| $\rho_{i1\leftrightarrow i2}$ *^d^* | 0.348 (0.318; 0.378) | - |
|  |  |  |
| RMSEA *^e^* | 0.048 (0.044; 0.052) | 0.053 (0.048; 0.057) |
|  |  |  |
| CFI *^f^* | 0.984 | 0.983 |
| TLI *^g^* | 0.978 | 0.976 |
| ^a^ Model with residual correlations between Items freely estimated; ^b^ Model with residual correlations specified in model 1 addressed; ^c^ Factor Loadings; ^d^ Residual Correlations; *^e^* Root-Mean-Square Error of Approximation (RMSEA) (90% Confidence Interval); *^f^* Comparative Fit Index (CFI)); *^g^* Tucker-Lewis Index (TLI). | | |

Supplementary Table 5. Confirmatory Factor Analysis of Module 04 of the Initial Child Development Observation Form from the Criança Feliz Program (partition 2).

| **Item** | **Fator** |
| --- | --- |
|  | ***λ_i_*_(f1)_ *^a^*** |
| i1 | 0.659 |
| i2 | 0.781 |
| i3 | 0.741 |
| i4 | 0.675 |
| i5 | 0.761 |
| i6 | 0.709 |
| i7 | 0.785 |
|  |  |
| RMSEA *^c^* | 0.052 (0.047; 0.056) |
| CFI *^d^* | 0.987 |
| TLI *^e^* | 0.980 |
| *^a^* Factor Loadings; *^b^* Root-Mean-Square Error of Approximation (RMSEA) (90% Confidence Interval); *^c^* Comparative Fit Index (CFI)); *^d^* Tucker-Lewis Index (TLI). | |

Supplementary Table 6. Confirmatory Factor Analysis of Module 05 of the Initial Child Development Observation Form from the Criança Feliz Program (partition 2).

| **Item** | **Model 1** *^a^* | **Model 2 *^b^*** |
| --- | --- | --- |
|  | ***λ_i_*_(f1)_ *^c^*** | ***λ_i_*_(f1)_ *^c^*** |
| i1 | 0.660 | - |
| i2 | 0.719 | 0.718 |
| i3 | 0.759 | 0.767 |
| i4 | 0.666 | 0.663 |
| i5 | 0.761 | 0.735 |
| i6 | 0.778 | 0.793 |
|  |  |  |
| $\rho_{i1\leftrightarrow i2}$ *^d^* | 0.549 (0.525; 0.572) |  |
|  |  |  |
| RMSEA *^e^* | 0.040 (0.035; 0.045) | 0.026 (0.020; 0.033) |
| CFI *^f^* | 0.995 | 0.998 |
| TLI *^g^* | 0.991 | 0.996 |
| ^a^ Model with residual correlations between Items freely estimated; ^b^ Model with residual correlations specified in model 1 addressed; ^c^ Factor Loadings; ^d^ Residual Correlations; *^e^* Root-Mean-Square Error of Approximation (RMSEA) (90% Confidence Interval); *^f^* Comparative Fit Index (CFI)); *^g^* Tucker-Lewis Index (TLI). | | |

Supplementary Table 7. Confirmatory Factor Analysis of Module 05 of Initial Child Development Observation Form from the Criança Feliz Program (partition 2).

| **Item** | **Modelo 1** *^a^* | **Modelo 2 *^b^*** |
| --- | --- | --- |
|  | ***λ_i_*_(f1)_ *^c^*** | ***λ_i_*_(f1)_ *^c^*** |
| i1 | 0.765 | 0.782 |
| i2 | 0.737 | 0.738 |
| i3 | 0.720 | 0.729 |
| i4 | 0.636 | - |
| i5 | 0.638 | 0.639 |
| i6 | 0.627 | - |
| i7 | 0.871 | 0.873 |
| i8 | 0.749 | 0.750 |
| i9 | 0.757 | 0.729 |
|  |  |  |
| $\rho_{i4\leftrightarrow i5}$ *^d^* | 0.372 (0.347; 0.398) | - |
| $\rho_{i4\leftrightarrow i6}$ *^d^* | 0.360 (0.336; 0.385) | - |
| $\rho_{i5\leftrightarrow i6}$ *^d^* | 0.453 (0.430; 0.476) | - |
|  |  |  |
| RMSEA *^d^* | 0.044 (0.041; 0.047) | 0.048 (0.045; 0.052) |
| CFI *^e^* | 0.986 | 0.985 |
| TLI *^f^* | 0.979 | 0.977 |
| ^a^ Model with residual correlations between Items freely estimated; ^b^ Model with residual correlations specified in model 1 addressed; ^c^ Factor Loadings; ^d^ Residual Correlations; *^e^* Root-Mean-Square Error of Approximation (RMSEA) (90% Confidence Interval); *^f^* Comparative Fit Index (CFI)); *^g^* Tucker-Lewis Index (TLI).  . | | |

Supplementary Table 8. Confirmatory Factor Analysis of Module 07 of Initial Child Development Observation Form from the Criança Feliz Program (partition 2).

| **Item** | **Model 1*^a^*** | **Model 2*^b^*** |
| --- | --- | --- |
|  | ***λ_i_*_(f1)_ *^c^*** | ***λ_i_*_(f1)_ *^c^*** |
| i1 | 0.814 | 0.820 |
| i2 | 0.701 | 0.689 |
| i3 | 0.790 | 0.794 |
| i4 | 0.813 | 0.829 |
| i5 | 0.718 | 0.706 |
| i6 | 0.677 | - |
| i7 | 0.707 | 0.703 |
| i8 | 0.813 | 0.823 |
| i9 | 0.823 | 0.846 |
| i10 | 0.673 | 0.836 |
| i11 | 0.738 | 0.651 |
| i12 | 0.609 | - |
|  |  |  |
| $\rho_{i6\leftrightarrow i7}$ *^d^* | 0.378 (0.350; 0.406) | - |
| $\rho_{i11\leftrightarrow i12}$ *^d^* | 0.412 (0.379; 0.444) | - |
|  |  |  |
| RMSEA *^e^* | 0.041 (0.039; 0.043) | 0.038 (0.036; 0.040) |
| CFI *^f^* | 0.974 | 0.979 |
| TLI *^g^* | 0.967 | 0.973 |
| ^a^ Model with residual correlations between Items freely estimated; ^b^ Model with residual correlations specified in model 1 addressed; ^c^ Factor Loadings; ^d^ Residual Correlations; *^e^* Root-Mean-Square Error of Approximation (RMSEA) (90% Confidence Interval); *^f^* Comparative Fit Index (CFI)); *^g^* Tucker-Lewis Index (TLI).  . | | |
